# Real‐world analysis of the clinical care pathway of patients living with Alzheimer’s disease in the United States according to stage of cognitive impairment at diagnosis

**DOI:** 10.1002/alz.084649

**Published:** 2025-01-09

**Authors:** Elnara Fazio‐Eynullayeva, Sarah Cotton, Eddie Jones, Sophie Kirkman, Paul Mystkowski, Soeren Mattke, Lei Lv

**Affiliations:** ^1^ Novo Nordisk Inc., Plainsboro, NJ USA; ^2^ Adelphi Real World, Bollington, Cheshire United Kingdom; ^3^ University of Southern California, Los Angeles, CA USA; ^4^ Novo Nordisk Inc, Plainsboro, NJ USA

## Abstract

**Background:**

We analyzed the clinical care pathway of patients from symptom development to diagnosis at varying stages of Alzheimer’s disease (AD) to understand the path and barriers to diagnosis.

**Methods:**

Data were from the US Adelphi Real World Dementia Disease Specific Programme™ (cross‐sectional survey of physicians and their patients with retrospective data collection) between December 2022 and September 2023. Physicians (primary care physicians [PCPs] and specialists) reported patient characteristics and disease severity, and patients reported their disease experience. Patients were aged ≥50 years with probable AD based on physician diagnosis and cognitive impairment severity assessed by Mini‐Mental State Examination (MMSE) scores: 26‒29, mild cognitive impairment (MCI) due to AD; 21‒25, mild AD dementia; 11‒20, moderate AD dementia; 0‒10, severe AD dementia.

**Results:**

Overall, 191 physicians (83 PCPs, 108 specialists) reported data for 870 patients. Mean (SD) age was 74.7 (8.4) years and 48.3% of patients were female. At initial diagnosis, 22% of patients had MMSE scores consistent with MCI due to AD, 49% mild AD dementia, 26% moderate AD dementia and 2% severe AD dementia.

Irrespective of AD severity, short‐term memory loss prompted first physician evaluation for 83% of patients. Most patients (53.1%) delayed evaluation because they perceived memory issues to be a normal part of aging.

Median (IQR) time between symptom onset and first evaluation was 17.3 (4.4;43.4) weeks, and time from first evaluation to diagnosis was 3.0 (0.0;13.1) weeks. Around half of patients (47.0%) were referred to a secondary physician before diagnosis, with median (IQR) time from first evaluation to referral of 8.7 (2.3;19.9) weeks. For referred patients, median (IQR) time from first evaluation to diagnosis was 9.9 (3.1;26.2) weeks.

In 75.3% of cases, patients first visited a PCP (range: 71.4‐76.8%, across severity subgroups). Specialists made 57.9% of initial diagnoses versus 41.8% by PCPs; 61.9% versus 38.1%, respectively, for MCI due to AD and 57.1% versus 42.9%, respectively, for severe AD dementia.

**Conclusion:**

While specialists and PCPs both diagnosed AD across all stages, specialists were more likely to diagnose patients in the MCI stage. Delays in AD diagnosis are common and new strategies to address these are warranted.

